# Pulse transit time respiratory swing as a diagnostic test for obstructive sleep apnoea in children—An observational study

**DOI:** 10.3389/fneur.2025.1632919

**Published:** 2025-09-22

**Authors:** Michael P. Yanney, Albert Essiam, Nicola J. Rowbotham, Andrew P. Prayle

**Affiliations:** ^1^Sherwood Forest Hospitals Foundation Trust, Sutton-in-Ashfield, Nottinghamshire, United Kingdom; ^2^Department of Data Science, Post University, Waterbury, CT, United States; ^3^Lifespan and Population Health, School of Medicine, University of Nottingham, Nottingham, United Kingdom; ^4^Nottingham NIHR Biomedical Research Centre, Nottingham University Hospitals NHS Trust, Nottingham, United Kingdom

**Keywords:** pulse transit time, sleep-disordered breathing, obstructive sleep apnoea, upper airway resistance syndrome, oximetry, children, machine learning

## Abstract

**Background:**

Pulse transit time (PTT) has been identified as a potentially useful tool for diagnosing obstructive sleep apnoea (OSA) due to its high sensitivity in detecting cortical arousals. However, its use in clinical practise has been disappointing as it appears to lack the ability to distinguish between individuals with or without OSA. The majority of studies evaluating PTT for sleep-disordered breathing (SDB) have assessed the pulse transit time arousal index (PTT-AI), and there are limited published data on PTT respiratory swing (PTTrs). We previously conducted an observational study of PTT in 368 children with SDB. Our findings indicated that depending on the cut-off used, PTTrs identified OSA with low-to-moderate sensitivity and moderate-to-high specificity, using limited multi-channel sleep studies (MCSS) as the comparator.

**Methods:**

We conducted this cross-sectional observational study in another cohort of 1,031 children with SDB who attended a secondary care centre consecutively for MCSS between July 2022 and November 2024. Polysomnography (PSG) is not available in UK secondary care centres, and our use of MCSS in this setting is novel. We analysed the data of 629 children using multinomial regression and machine learning.

**Results:**

We found a stepwise increase in PTTrs with increasing severity of SDB. Children with mild OSA had a mean PTTrs of 20.7 ms. Machine learning analysis indicated that the oxygen desaturation index (ODI3) and PTTrs were the most important predictors of SDB amongst the 15 variables studied.

**Conclusion:**

Our findings suggest that PTTrs could complement oximetry to improve the detection of OSA in children. A validation study comparing PTTrs with PSG is needed.

## Introduction

Polysomnography (PSG) is the gold standard for diagnosing obstructive sleep apnoea (OSA). However, due to limited availability, there has been a reliance on oximetry or management based solely on clinical evaluation in secondary care centres across the UK (1, 2). A systematic review shows that oximetry can accurately detect children with moderate or severe OSA but has low sensitivity for detecting mild OSA in children with comorbidities (3). Pulse transit time (PTT) has been evaluated for its ability to provide a non-invasive measure of arousals and respiratory effort in children with sleep-disordered breathing (SDB) (4–8). However, its use in clinical practise has been disappointing, largely because the PTT arousal index (PTT-AI) appears to lack the ability to effectively discriminate between children with OSA and those without OSA.

We previously reported the results of an observational study on the use of PTT in children with SDB. The study showed that the PTT respiratory swing (PTTrs) can effectively detect mild OSA or upper airway resistance syndrome (UARS) diagnosed through video assessment in children who had normal or inconclusive oximetry results (9). PTTrs measures the size of the oscillations in PTT during inhalation and has been shown to correlate closely with the degree of inspiratory effort (10). There is minimal published data on PTTrs, and following a systematic review of the literature, we identified only four other studies that report on this index (also referred to as PTT swing, PTT inspiratory effort, or ΔPTT) in children with SDB (4, 10–13). To investigate the diagnostic accuracy of PTTrs, it is essential to compare the results with PSG. Before designing this study, we conducted an observational study on PTT data in a larger independent cohort of children (approximately double the previous sample size) to assess the reproducibility of the previous PTT findings and to identify mean or threshold PTTrs values with better precision.

There has been considerable recent interest in utilising machine learning (artificial intelligence) to analyse datasets. This approach provides new insights into previously unrecognised associations between variables and outcomes of interest (14, 15). In addition to conventional statistics, we used multinomial regression and supervised machine learning to analyse associations between PTT, oximetry, or other variables of interest and a diagnosis of OSA or UARS in children.

## Methods

We conducted a cross-sectional observational study using data from 1,031 children (< 18 years of age) recorded in our sleep study database, who were referred consecutively to a UK secondary care centre for sleep studies between July 2022 and November 2024. The sleep study database was maintained for audit and service evaluation purposes. Children were referred with symptoms of SDB by otorhinolaryngologists, paediatricians, or general practitioners. We performed multi-channel sleep studies (MCSS) using two Stowood Scientific Instruments VISI-3 sleep systems, incorporating electrocardiography (ECG), video, sound, movement, pulse transit time, and oximetry data. MCSS resemble cardiorespiratory studies (CRSS) or respiratory polygraphy, but do not fulfil the criteria for CRSS as they do not measure airflow, and therefore cannot report an apnoea-hypopnea (AHI) index. PSG is not available in UK secondary care centres and so was not available for use in this study. The VISI-3 sleep system utilises Masimo technology to obtain oximetry data with averaging times of 2–4 s. Oximetry was measured with probes attached to either a finger or a toe. The video was recorded using a Sony EVI-D90P infra-red camera (https://pro.sony/en_GB/products/ptz-network-cameras/evi-d90-d90p-pal-).

**Figure 1:**
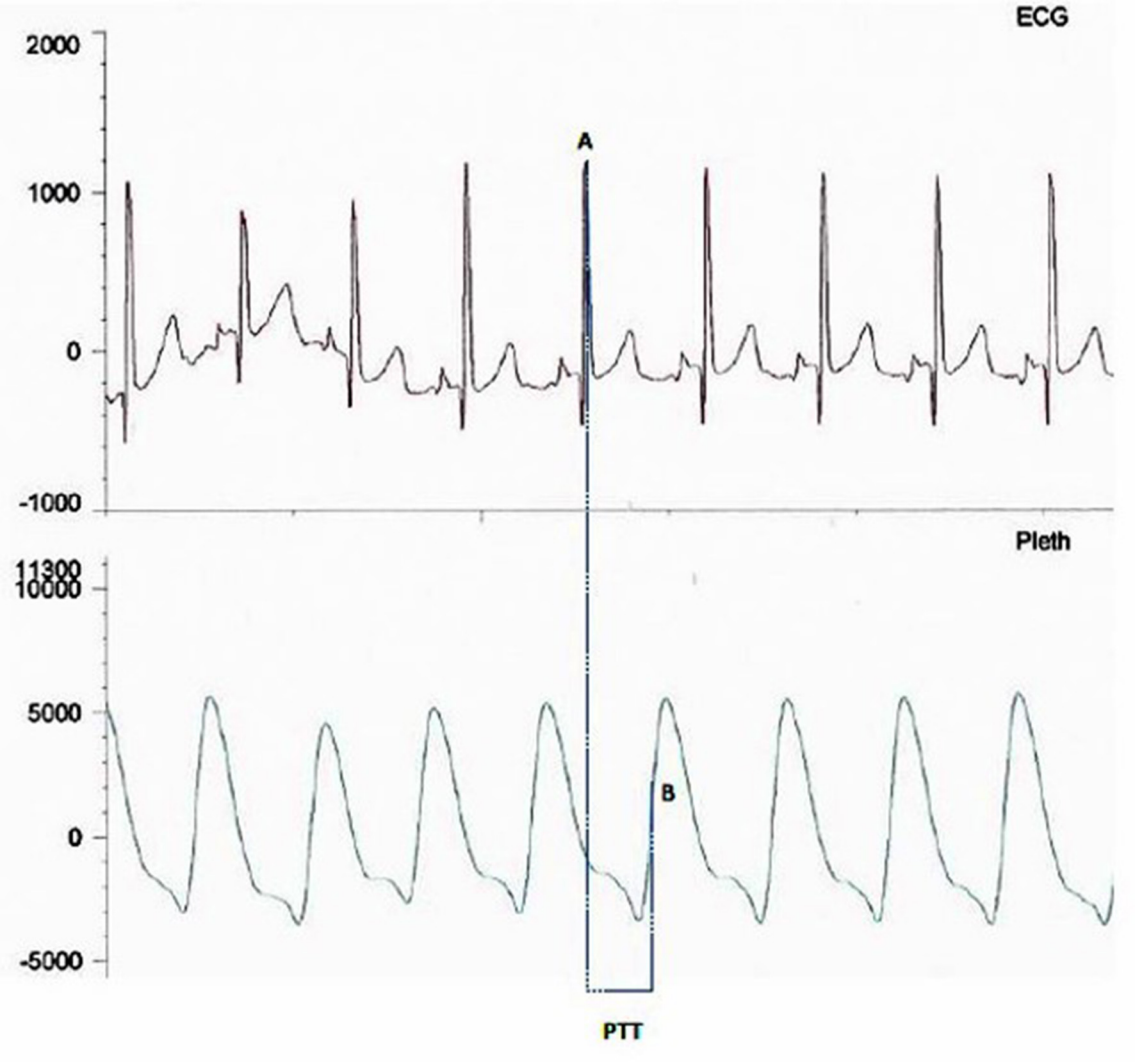
Image showing PTT measurement between the mid-point of the R wave **(A)** on the ECG trace and the mid-point of the up slope **(B)** on the plethysmography curve.

**Figure 2:**
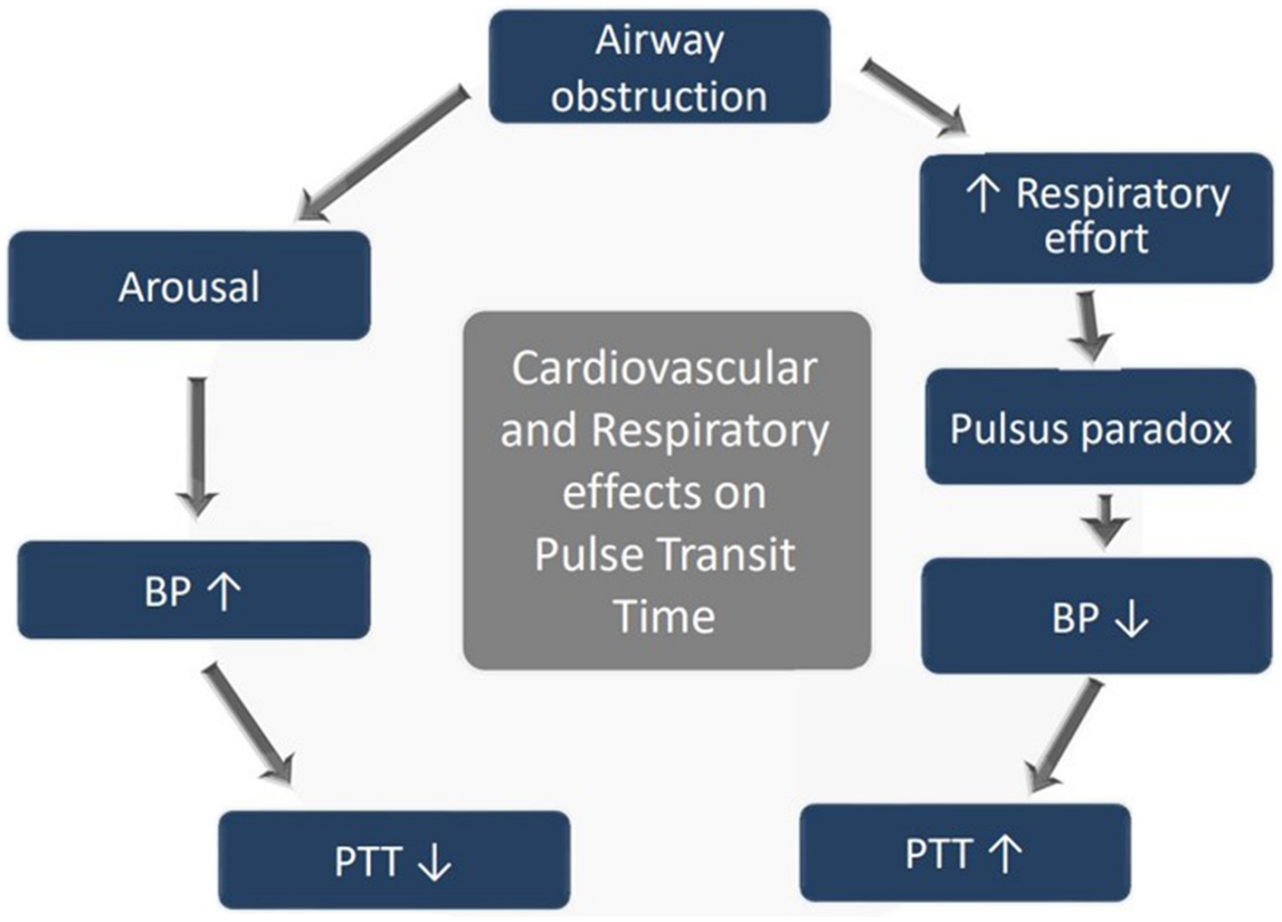
Diagram showing cardiovascular and respiratory effects on PTT.

PTT is measured from the mid-point of the R wave of the QRS sequence obtained by ECG to a pulse waveform value obtained from an oximeter and estimated at 50% of the maximum point of the plethysmography curve (Figure 1). A rise in mean arterial pressure (MAP) causes the pulse wave to travel faster and PTT to shorten; conversely, a lower MAP causes PTT to lengthen. PTT is therefore an estimate of the time it takes for the pulse pressure wave to travel from the aortic valve to the periphery and is inversely correlated to blood pressure (BP) changes (4). PTT can identify changes in inspiratory effort because of BP fluctuations induced by negative pleural pressure swings. Figure 1 illustrates how PTT is measured, and Figure 2 presents a diagram illustrating cardiovascular and respiratory effects on PTT.

PTT is measured in millisecond and calculated with the VISI-3 software. PTT2 is calculated by the software which applies a 17-point averaging (approximately 3.5 s) to the recorded raw channel PTT data. A PTT arousal was calculated as a drop in PTT2 ≥ 15 ms within 5–45 s if the PTT2 value was in the valid range of 150–500 ms. The PTT arousal index (PTT-AI) was calculated as the number of PTT arousals/h over the study duration. The average respiratory swing (PTTrs) was analysed using a derived PTT channel, which had been interpolated for 1 s, and then a 3-sample moving window average was applied. The PTTrs is the average size of the respiratory rise from an inspiratory trough to expiratory peak and is measured in millisecond (ms).

### Exclusions

**Figure 3:**
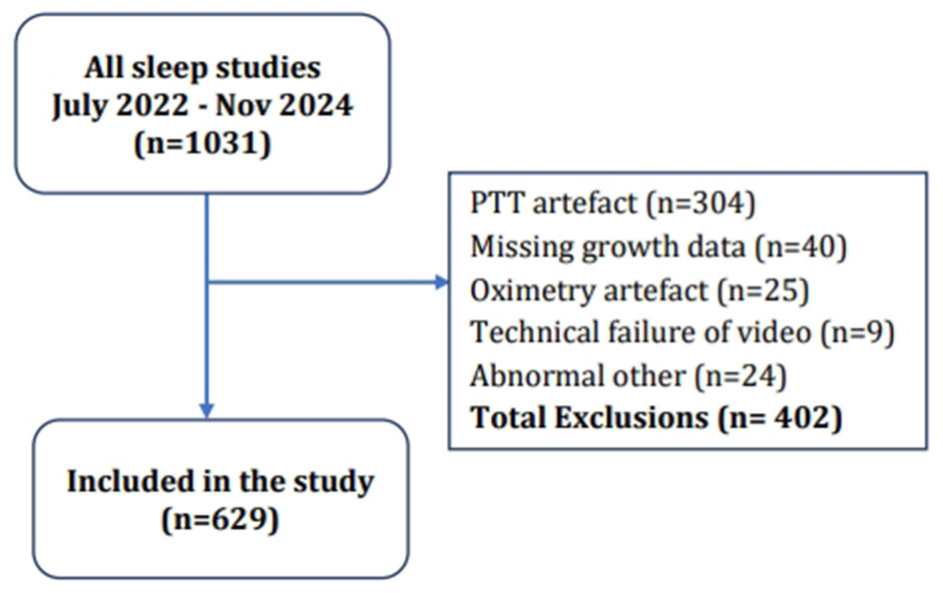
Flow diagram of study participants and reasons for exclusion.

The PTT and oximetry traces were assessed for artefact by a sleep physiologist before reporting. Oximetry artefacts associated with movement or low perfusion were excluded manually in each 20-min epoch. The PTT artefact was also manually excluded using a 12 h, full study PTT trace by a physiologist. Children with < 4 h of artefact-free oximetry data were excluded in line with current guidance (3). There is no guidance for the acceptable minimum duration of PTT data required for analysis. However, in line with our previous study, we excluded sleep studies with < 3 h of artefact-free PTT data. We chose this duration as it represents 75% of the accepted minimum length of an oximetry study and is a compromise value to avoid excluding an even larger proportion of studies due to PTT artefact. PTT is especially prone to artefacts resulting from either photoplethysmography or electrocardiography (ECG) signal dropout. The PTT artefact was identified manually as rapid spikes in PTT exceeding 50 millisecond (ms); typically, these were >100 ms. Further details on PTT artefact removal are provided in our previous study and are illustrated in Figure A1 (Appendix). We excluded children who had abnormal sleep study findings due to causes unrelated to OSA. They were categorised as “abnormal other,” and included central apnoeas, neonatal chronic lung disease, or lower respiratory tract infection. We excluded children with incomplete data, which prevented the inclusion of their data in the multinomial regression or machine learning analysis. Reasons for exclusion are shown in Figure 3.

**Figure 4:**
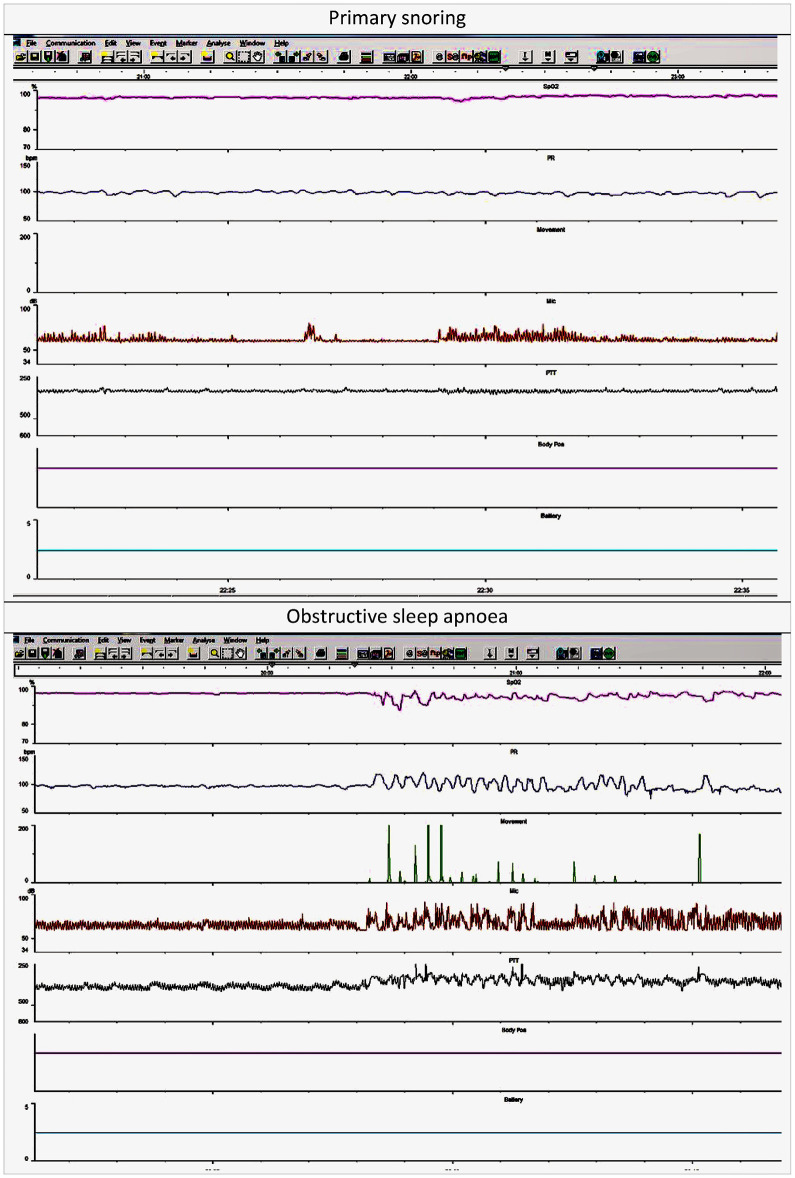
Example traces obtained using MCSS in a child with primary snoring and another with obstructive sleep apnoea.

Sleep study categories were determined by a single clinician (MY) assisted by a physiologist supporting the sleep reporting session, using oximetry, video, and sound criteria, as listed below. Inter-rater or intra-rater reliability data were not obtained. The oximetry, heart rate, sound, and movement traces were used to identify sections of video that needed closer inspection (Figure 4). As defined below, the video was then assessed for evidence of obstructive episodes. Following assessment of the oximetry data, sleep study montage, and the video, the reporting clinician assigned one of the following five categories: normal; primary snoring; upper airway resistance syndrome; obstructive sleep apnoea or abnormal other. The reporting clinician remained blind to PTT values until after the sleep study categories had been determined. Our sleep reporting methods are described in more detail elsewhere (9, 16).

The children's age, gender, referring clinician, oximetry indices, pulse transit time indices (PTTrs, PTT-AI), duration of artefact-free oximetry data, duration of artefact-free PTT data, and growth measurements were recorded in a database maintained for audit and service evaluation purposes. The following oximetry indices were recorded: mean saturation, minimum saturation, mean desaturation nadir, dip index (ODI4) defined as >4 % drop (i.e., 5% or greater), and dip index (ODI3) defined as > 3% drop (i.e., 4% or greater) in baseline saturation/h and lasting for >5 but < 180 s. An oximetry score of 1–4 was assigned based on the criteria listed below. We used a combination of McGill's oximetry criteria for describing OSA severity (17) and ODI3 or ODI4 thresholds as recommended in the BTS guidelines for paediatric sleep-disordered breathing (3).

**Oximetry scores:**

(1) Normal or inconclusive oximetry defined using McGill's criteria (17).

(2) ODI3 ≥ 7 or ODI4 ≥ 4 and minimum oxygen saturation of ≥80%.

(3) ODI3 ≥ 7 or ODI4 ≥ 4 and < 3 minimum oxygen saturation episodes < 80%.

(4) ODI3 ≥7 or ODI4 ≥ 4, and ≥ 3 minimum oxygen saturation episodes < 80%.

**Sleep study category definitions:**

- **Normal**: No snoring or obstructed breathing evident on video and oximetry score of 1.
- **Primary snoring**: Snoring but < 3 witnessed obstructive episodes on video and an oximetry score of 1.
- **UARS/mild OSA:** Video and sound evidence of 3 or more discrete periods of obstructed breathing, associated arousals, and an oximetry score of 1.
- **Moderate OSA**: Video and sound evidence of obstructed breathing, associated arousals, and an oximetry score of 2 or 3.
- **Severe OSA**: Video and sound evidence of obstructed breathing, associated arousals, and an oximetry score of 4.
- **Abnormal other**: An oximetry score ≥ 2 but with no video evidence of obstruction.

**Other definitions:**

- **Obstructive episodes** were identified on the video when there was a pause in snoring but continued respiratory effort, followed by an airway opening noise and an arousal.
- **An arousal** was identified if there was a movement associated with an obstructive episode and a corresponding pulse rate rise.

Weight was measured with SECA (Hamburg, Germany) electronic chair scales or SECA baby scales. Height was measured using a SECA wall-mounted stadiometer or a Dunmow Rollameter (Harlow Healthcare, UK).

### Data analysis

We analysed the data of children with complete datasets during the period from July 2022 to November 2024. Microsoft Excel and the R program were used to calculate summary statistics for quantitative data, including the mean, median, range, and standard deviation (SD).

We conducted two types of analysis to understand the impact of independent variables on the sleep study diagnoses (scores). First, we used multinomial regression to understand the sensitivity of a 1% change in an independent variable on the diagnostic score. Due to the probabilistic nature of the impact, changes in the diagnostic score are expressed in log odds ratios. Multinomial regression equations are used to model the diagnostic score as a function of the independent variables. The regression equations have the form:


(1) $$\begin{array}{l} \ln\left(\frac{p\left(Score=1\right)}{p\left(Score=3\right)}\right)=\\ Intercept\;1+C_{1}\;PTTSwing+C_{2}\;Sex+C_{3}\;Mean.Nadir\\ +C_{4}\;Age_{mth}+C_{5}\;Oximetry+\;C_{6}\;Weight\\ +C_{7}\;Height+C_{8}\;ODI4 \end{array}$$

(2) $$\begin{array}{l} \ln\left(\frac{p\left(Score=2\right)}{p\left(Score=3\right)}\right)=\\ Intercept\;2+D_{1}\;PTTSwing+D_{2}\;Sex+D_{3}\;Mean.Nadir\\ +D_{4}\;Age_{mth}+D_{5}\;Oximetry+\;D_{6\;}Weight\\ +D_{7}\;Height+D_{8}\;ODI4 \end{array}$$

The C and D coefficients provided in Equations 1, 2 are calculated by the R program.

- C_1_ A one-unit increase in the variable PTTrs is associated with a decrease in the log odds of being in Score = 1 vs. Score = 3 in the amount of 0.02.
- C_2_ A one unit increase in the variable Sex = M is associated with a decrease in the log odds of being in Score 1 vs. Score = 3 in the amount of 0.05.

For the second analysis, we used machine learning to model the sleep study diagnostic scores as a function of the independent variables: PTTrs, PTT-AI, PTT duration, oximetry score, ODI3, ODI4, minimum saturation, average saturation, mean desaturation nadir, study duration, gender, weight, height, heart rate and heart rate standard deviation. For the machine learning analysis, we used a logistic regression model, which had the form:


$$ln\left(\frac{p}{1-p}\right)=\sum_{i-1}^{n}\left(\beta_{0}+\beta_{i}X_{i}\right)$$

where

*p* = probability of having a sleep disorder, 1 for a disorder, 0 otherwise;

β = coefficient to be determined from the model; and

*X* = an independent variable such as weight, age, etc.

Children with missing data were excluded from the study; therefore, the machine learning analysis was only performed on those with complete datasets. No data were normalised or scaled. We performed a second type of machine learning analysis using a tree-based decision model, and the results of this analysis are included in the Appendix. For the logistic regression model analysis, we used the entire dataset, as this model is more accurate with a larger dataset. For the decision tree model in machine learning analysis, the data were split into training and testing sets to further improve the accuracy of this model.

### Ethical considerations

The Research and Innovation department at Sherwood Forest Hospitals Foundation Trust was approached and confirmed that ethical approval was not required for the analysis of anonymised sleep study data, which involved no change to usual care (18).

## Results

The flow through the study is illustrated in the Consort chart (Figure 3). Complete data were available for 629 children (336 male and 293 female children) and used for the analysis. The mean age of the children in the study was 6.5 years (SD 3.1; 0.6–17.6). Table 1 lists the demographics and sleep study diagnoses of children included in the study and Table 2 lists demographics and sleep study diagnoses of children who were excluded.

**Table 1:** Demographics and sleep study findings of children included in the study.

**Total (** ***n*** **)**		**All**		**Normal study**		**Primary snoring**		**UARS/mild OSA**		**Moderate OSA**		**Severe OSA**	
		**629**		**174**		**239**		**132**		**63**		**21**	
Sex (*n*)	Male	336	53%	83	48%	140	59%	69	52%	32	51%	12	57%
	Female	293	47%	91	52%	99	41%	63	48%	31	49%	9	43%
Age (years)	Mean (SD)	6.5	3.1	6.8	3.2	7.0	3.2	5.8	2.6	5.8	3.1	4.3	1.5
	Median (range)	5.8	0.6–17.6	6.2	1.4–17.6	6.2	1.3–17.3	5.5	0.6–16.4	5.0	2.1–17.2	4.5	2–7.8
Weight (kg)	Mean (SD)	27.3	16.7	27.9	15.0	30.1	18.3	23.3	13.7	26.1	20.0	18.8	6.6
	Median (range)	21.4	8.8–128.7	23.5	10.6–99	23.0	10.2–120.3	19.5	10.8–86.3	18.9	11.3–128.7	16.5	8.8–39.3
Height (cm)	Mean (SD)	118.8	20.3	120.9	20.0	122.0	20.8	114.2	18.2	115.3	21.4	105.2	13.5
	Median (range)	115.7	74.4–189	118.5	80–167.1	118.1	74.4–184.5	111.8	79–178.3	111.0	83.2–189.0	103.7	77.5–130
Referrer (*n*)	ENT	510	81%	134	77%	194	81%	114	86%	51	81%	17	81%
	PAED	116	18%	39	22%	44	18%	18	14%	11	17%	4	19%
	GP	3	0%	1	1%	1	0%	0	0%	1	2%	0	0%

**Table 2:** Demographics and sleep study findings of children excluded from the study.

**Total (** ***n*** **)**		**All**		**Normal study**		**Primary snoring**		**UARS/mild OSA**		**Moderate OSA**		**Severe OSA**		**Abnormal other**		**Technical failure**	
		**402**		**98**		**139**		**45**		**43**		**19**		**25**		**33**	
Sex (*n*)	Male	173	43%	59	60%	79	57%	27	60%	26	60%	12	63%	14	56%	12	36%
	Female	229	57%	39	40%	60	43%	18	40%	17	40%	7	37%	11	44%	21	64%
Age (years)	Mean (SD)	5.8	3.3	6.1	3.4	6.2	3.5	5.5	2.1	5.5	3.2	5.2	2.7	5.6	4.7	5.0	2.6
	Median (range)	5.3	0.2–17.1	5.3	0.24–16	5.5	0.3–16.9	5.6	1.4–11.9	5.0	0.5–17.1	5.1	1.8–11.6	4.1	0.3–16.5	5.0	0.4–11.6
Weight (kg)	Mean (SD)	26.6	19.1	25.9	16.7	28.3	21.7	24.5	14.4	28.9	23.3	23.8	15.4	25.9	21.3	22.5	9.7
	Median (range)	19.7	5.6–134.1	20.0	5.6–100	19.5	8.4–134.1	20.8	10.2–96.3	20.8	7.8–109.1	17.5	8.6–61.7	15.5	5.7–87.2	22.1	12.6–52
Height (cm)	Mean (SD)	115.9	22.6	116.8	23.3	118.5	23.1	115.6	16.5	115.1	22.9	110.9	22.3	108.3	29.3	109.8	18.0
	Median (range)	113.2	63–198	113.2	66.5–173.4	114.2	79.8–198	114.1	74–163	112.6	70–168.3	109.5	76.7–154.5	99.2	63–158.9	111.3	82.8–141.5
Referrer (*n*)	ENT	290	72%	68	69%	101	73%	34	76%	34	79%	17	89%	12	48%	24	73%
	PAED	108	27%	29	30%	37	27%	10	22%	8	19%	2	11%	13	52%	9	27%
	GP	4	1%	1	1%	1	1%	1	2%	1	2%	0	0%	0	0%	0	0%

### Comorbidities

Children with comorbidities comprised approximately 5% of the total study cohort (*n* = 31). There were 16 children with obesity defined as a body mass index (BMI) > 2 standard deviation scores above the mean. Four children had Down syndrome, and another three had other chromosomal abnormalities associated with abnormalities of tone and/or development, such as deletion of chromosome 6, duplication of the long arm of chromosome 6, and Potocki-Lupski Syndrome. Six children had neurodisability or neuromuscular disorders: four children had cerebral palsy with developmental delay and a Gross Motor Function Classification System (GMFCS) level 1 or greater associated with premature delivery; 1 child had a neuronal migration defect and associated developmental delay; another child had congenital myopathy. Two children had structural airway abnormalities: micrognathia and laryngomalacia.

**Figure 5:**
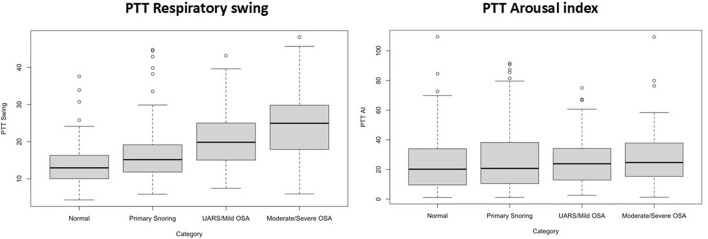
Box plots of PTT respiratory swing and PTT arousal index according to sleep study diagnosis.

**Figure 6:**
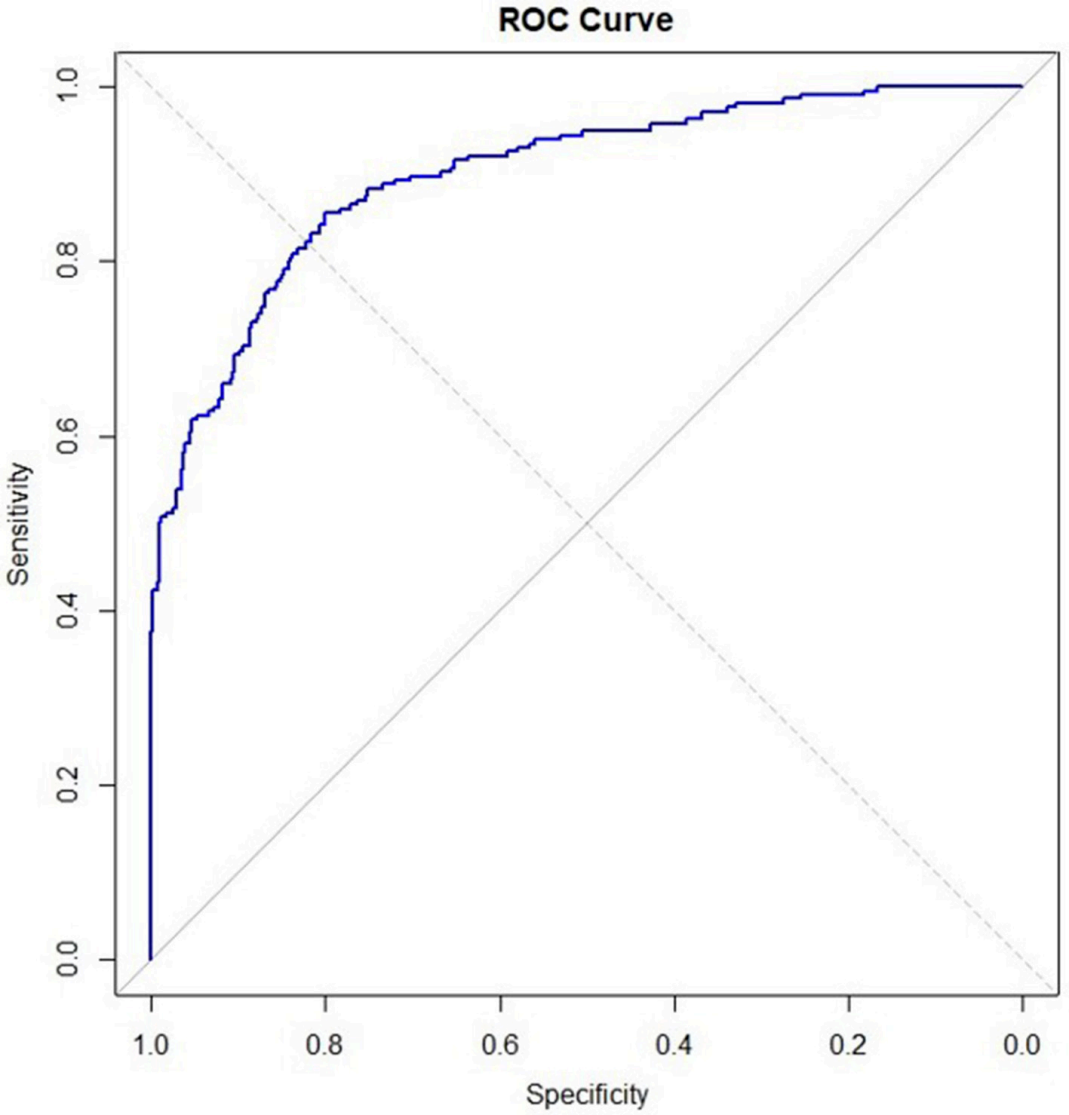
Receiver operator characteristic curve obtained with logistic regression model using the six most significant variables (ODI3, PTTrs, Average SpO_2_, Average heart rate, PTT-AI, and PTT duration), showing the model's diagnostic performance, with specificity on *x*-axis and sensitivity on *y*-axis. Area under the curve (AUC) is 0.899.

**Figure 7:**
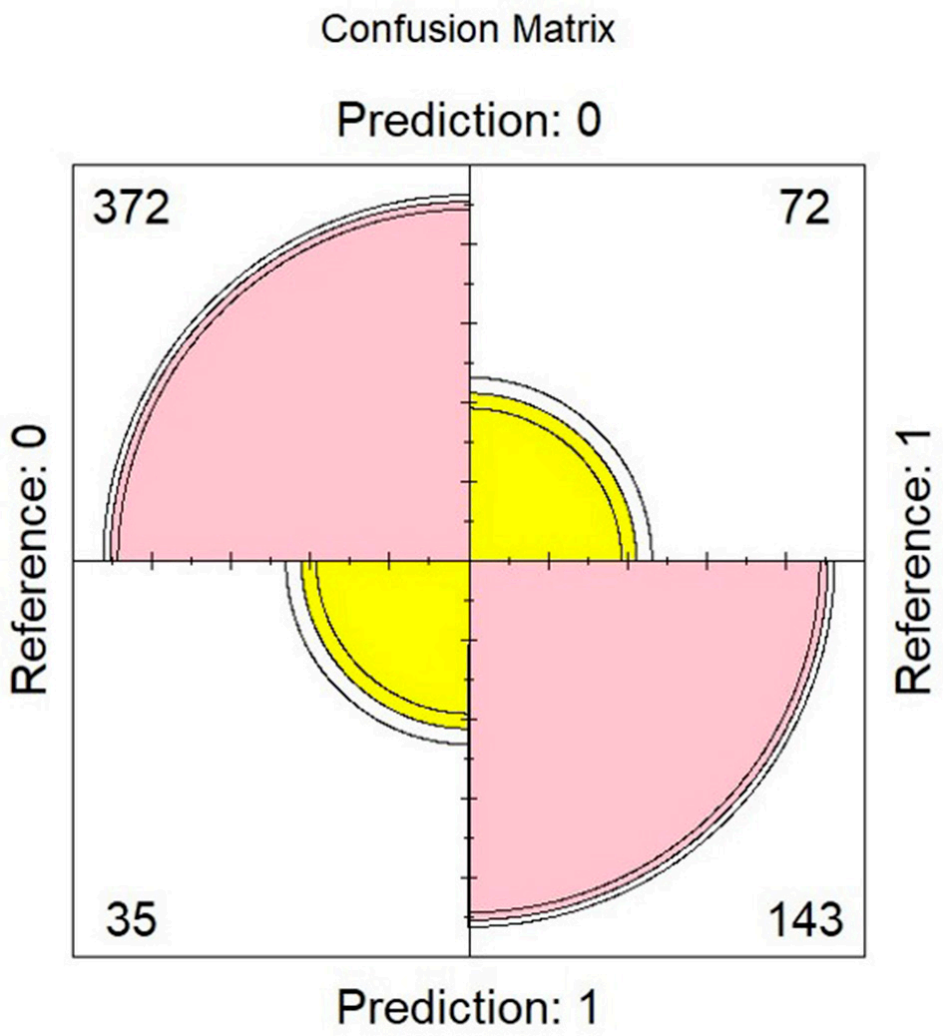
Confusion matrix obtained using a logistic regression model based on 15 variables, showing true negatives (*n* = 372), true positives (*n* = 143), false positives (*n* = 35), and false negatives (*n* = 72). Reference = diagnosis based on reference test (MCSS); predicted = diagnosis predicted by the model. Diagnostic performance metrics are shown in Table 7. Other logistics regression results are in Table A1.

**Figure 8:**
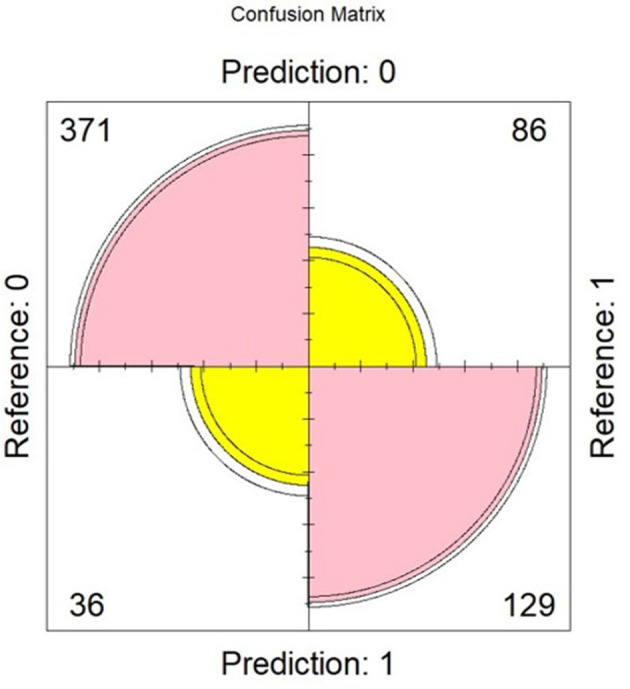
Confusion matrix obtained using the logistic regression model and based on ODI3 and PTTrs (using subjects with ≥ 3 h of PTT data), showing true negatives (*n* = 371), true positives (*n* = 129), false positives (*n* = 36), and false negatives (*n* = 86). Reference = diagnosis based on reference test (MCSS); predicted = diagnosis predicted by the model. Model performance metrics are shown in Table 8.

**Figure 9:**
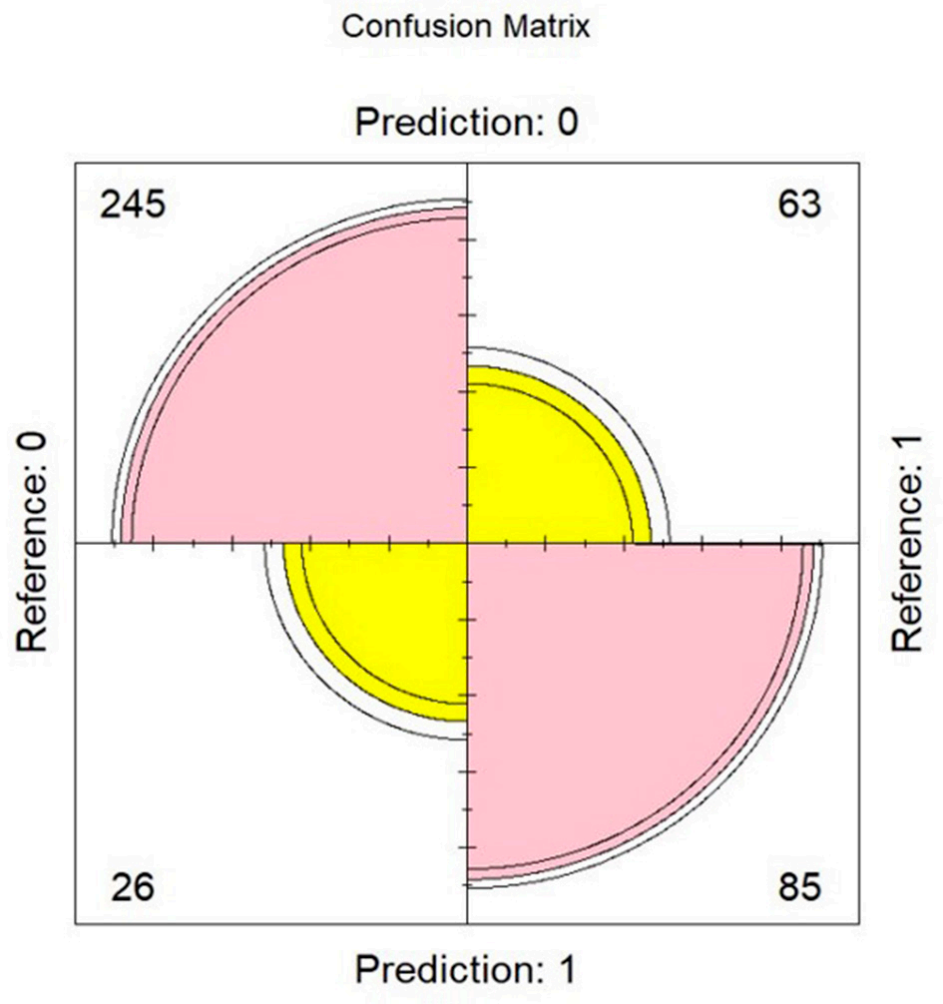
Confusion matrix obtained using a logistic regression model and based on ODI3 and PTTrs (using subjects with ≥ 4 h of PTT data), showing true negatives (*n* = 245), true positives (*n* = 85), false positives (*n* = 26), and false negatives (*n* = 63). Reference = diagnosis based on reference test (MCSS); predicted = diagnosis predicted by the model. Model performance metrics are shown in Table 9.

Tables 3, 4 present the results of the multinomial regression analysis and the significance values of PTTrs, and age according to sleep study diagnosis. From the initial regression analysis of all 15 variables, the insignificant variables (*p*-values > 0.05) were as follows: oximetry score, weight, height, heart rate standard deviation, minimum saturation, mean desaturation nadir, ODI4, study duration, and gender. The remaining six variables with *p*-values < 0.05; ODI3, mean saturation, mean heart rate, PTTrs, PTTAI, and PTT duration were evaluated in iteration 2 of the logistic regression model, and the results are shown in Table 5. A third iteration of the model identified ODI3 and PTTrs as the principal variables responsible for the model accuracy. Table 6 lists the results of the binomial model (using ODI3 and PTTrs). Table 7 lists the diagnostic performance statistics of the machine learning analysis using all 15 variables, and Table 8 shows the performance metrics of the model using ODI3 and PTTrs in children with a minimum of 3 h of artefact-free PTT data. Table 9 lists the performance metrics for the binomial model (ODI3 and PTTrs), but only in children who had a minimum of 4 h of PTT data available. Table 10 is a summary of the mean PTT duration with SD according to sleep study categories, showing a comparison of the numbers and proportions of children who had a minimum of 3 or 4 h of artefact-free PTT data available. Figure 5 is a box plot of the PTTrs and PTT-AI values according to sleep study diagnosis. Figure 6 is a receiver operating characteristic curve illustrating the performance of the machine learning model as a diagnostic test, with an area under the curve (AUC) of 0.899. Figures 7, 8 are confusion matrices that demonstrate the performance of the machine learning analysis based on the use of all 15 variables and the 2 principal variables (ODI3 and PTTrs). Figure 9 is a confusion matrix of ODI3 and PTTrs, but only including children with a minimum PTT duration of 4 h. Other results of the machine learning analysis are presented in the Appendix, including results of a decision tree-based machine learning analysis of the principal variables ODI3 and PTTrs.

**Table 3:** Mean age (months) according to sleep study findings assessed with multinomial regression.

**Diagnosis**	**Number (*n*)**	**Mean age (months)**	**SD**
Normal study/Primary snoring	413	82.86	38.0
UARS/OSA	216	67.73	32.1
**Statistics**			
Difference	−15.13		
Standard error	3.029		
95% C I	−21.08 to −9.18		
*t*-statistic	−4.995		
DF	627		
Significance level	*P* < 0.0001		

The populations are statistically different. A younger child is more likely to have OSA or UARS and the converse is true.

**Table 4:** Mean PTTrs according to sleep study diagnosis assessed with multinomial regression.

**Diagnosis**	**Number (*n*)**	**Mean PTTrs**	**SD**
Normal study	174	13.68	5.16
Occasional snoring	157	15.16	5.73
Regular snoring	82	18.65	7.34
UARS/Mild OSA	132	20.73	7.31
Moderate OSA	63	23.69	8.05
Severe OSA	21	26.68	9.07
Normal study or Primary snoring	413	15.23	6.14
UARS/OSA	216	22.17	7.93
**Statistics**			
Difference	6.940		
Standard error	0.571		
95% C I	5.8185–8.0615		
*T*-statistic	12.152		
DF	627		
Significance level	*P* < 0.0001		

There is a clear indication that PTTrs for children with a normal study or primary snoring is very distinct from those with UARS/OSA. The delta is 6.94 with a certainty of 100–0.0001%.

**Table 5:** Results of logistic regression model based on the 6 most significant variables.

**Regression metric**	**Coefficient from regression analysis**	**Standard error**	***Z*-value**	**Pr(>[*z*])**
Intercept	−53.91239	16.65653	−3.237	0.001209^**^
ODI3	0.68497	0.07841	8.736	< 2e-16^***^
Average SpO2	45.32684	16.88343	2.685	0.007260^**^
Average heart rate	0.04381	0.01212	3.616	0.000299^***^
PTTAI	−0.04646	0.00933	−4.979	6.39e-07^***^
PTTrs	0.15636	0.02215	7.061	1.65e-12^***^
PTT duration	0.31145	0.09568	3.255	0.001133^**^

Significance codes: 0 “^***^” 0.001 “^**^” 0.01 “^*^” 0.05 “.” 0.1 “ ” 1 (Dispersion parameter for binomial family taken to be 1).

Null deviance: 802.03 on 621 degrees of freedom.

Residual deviance: 448.95 on 615 degrees of freedom.

AIC: 462.95.

**Table 6:** Results of logistic regression binomial model based on ODI3 and PTTrs.

**Regression metric**	**Coefficient from regression analysis**	**Standard error**	***Z*-value**	**Pr(>[z])**
Intercept	−4.40943	0.37017	−11.912	< 2e-16^***^
ODI3	0.56794	0.06109	9.297	< 2e-16^***^
PTTrs	0.09504	0.01628	5.837	5.31e-09^***^

^***^ indicates a significance level of *p* < 0.001.

**Table 7:** Statistics showing the diagnostic performance of logistic regression analysis based on a model using 15 variables.

**Regression metric**	**Value from regression analysis**
Accuracy	0.828
95% CI	(0.7960, 0.8578)
No information rate	0.6543
*P*-Value [Acc > NIR]	< 2.2e-16
Kappa	0.6036
Mcnemar's test *P*-value	0.0005009
Sensitivity	0.9140 = 91.4%
Specificity	0.6651 = 66.51%
Pos pred value	0.8378=83.78%
Neg pred value	0.8034 =80.34%
Prevalence	0.6543 =65.43%
Detection rate	0.5981 =59.81%
Detection prevalence	0.7138 =71.38%
Balanced accuracy	0.7896 =78.96%

Statistics associated with Confusion matrix in Figure 7.

**Table 8:** Statistics showing diagnostic performance of logistic regression analysis based on a binomial model (ODI3 and PTTrs) in children with at least 3 h PTT data.

**Regression Metric**	**Value from regression Analysis**
Accuracy	0.8039
95% CI	(0.7704, 0.8344)
No information rate	0.6543
*P*-value [Acc > NIR]	< 2.2e-16
Kappa	0.5412
Mcnemar's test *P*-value	9.154e-16
Sensitivity	0.9115 = 91.15%
Specificity	0.6000 = 60.00%
Pos pred value	0.8118=81.18%
Neg pred value	0.7818 =78.18%
Prevalence	0.6543 =65.43%
Detection rate	0.5965 =59.65%
Detection prevalence	0.7347 =73.47%
Balanced accuracy	0.7558 =75.58%

Statistics associated with Confusion matrix in Figure 8.

**Table 9:** Statistics showing diagnostic performance of logistic regression analysis based on a binomial model (ODI3 and PTTrs) in children with at least 4 h PTT data.

**Regression metric**	**Value from regression analysis**
Accuracy	0.7876
95% CI	(0.7453, 0.8258)
No information rate	0.6468
*P*-value [Acc > NIR]	< 2.48e-10
Kappa	0.5072
Mcnemar's test *P*-value	0.0001356
Sensitivity	0.9041 = 90.41%
Specificity	0.5743 = 57.43%
Pos pred value	0.7955=79.55%
Neg pred value	0.7658 =76.58%
Prevalence	0.6468 =64.68%
Detection rate	0.5847 =58.47%
Detection prevalence	0.7351 =73.51%
Balanced accuracy	0.7392 =73.92%

Statistics associated with Confusion matrix in Figure 9.

**Table 10:** Number and proportion of children within sleep study categories with a minimum PTT duration of either 3 or 4 h and mean PTT duration with standard deviation.

**Minimum PTT duration**	**All**	**Normal study**	**Primary snoring**	**UARS/mild OSA**	**Moderate/severe OSA**
Number of children with PTT duration ≥ 3 h (%)	629 (100%)	174 (27.7%)	239 (38%)	132 (21%)	84 (13.3%)
Mean PTT duration in hours [SD]	4.79 [1.32]	4.64 [1.28]	4.88 [1.70]	4.88 [1.29]	4.86 [1.42]
Number of children with PTT duration ≥ 4 h (%)	419 (100%)	105 (25.1%)	166 (39.6%)	91 (21.7%)	57 (13.6%)
Mean PTT duration in hours [SD]	5.45 [1.13]	5.38 [1.14]	5.43 [1.13]	5.52 [1.03]	5.50 [1.28]

Mean oximetry duration: 8.61 h; SD 1.37.

## Discussion

Our results show a stepwise increase in the mean PTTrs from children with a normal sleep study to those with severe OSA. The results are consistent with our previous study findings, but are obtained using a different analysis, in a larger sample size, and show differences between the diagnostic categories with better precision (9). The multinomial regression analysis shows that the PTTrs findings are highly significant which suggests the observation is unlikely to be a chance finding. Our study also provides evidence of how machine learning analysis of large datasets can offer additional useful insights in clinical research. We initially anticipated that machine learning analysis would provide information on the relative importance or usefulness of different variables in our dataset. However, analysis undertaken to demonstrate performance metrics and model validation of the machine learning model identified that only 6 of the 15 variables assessed made a significant contribution to the model. By re-running the logistics regression model with the 6 most significant variables, we found that the model accuracy was effectively unchanged at 0.82, sensitivity 0.91, and specificity 0.66 compared with an accuracy of 0.83, sensitivity 0.91, and specificity 0.66 obtained with all 15 variables. To further identify the most parsimonious model, we selected the two variables with the highest level of significance (ODI3 and PTTrs) and re-ran the logistics regression analysis with these variables. As a result of this analysis, we identified that ODI3 and PTTrs were the principal variables in our model, achieving a model accuracy of 0.80, sensitivity of 0.91, and specificity of 0.60. We further validated the findings, confirming ODI3 and PTTrs as principal variables, using a decision tree-based machine learning model which also had a model accuracy of 0.80, sensitivity of 0.91, and specificity of 0.60 for predicting children with OSA or UARS. We believe that the concordance of the diagnostic accuracy results with two different machine learning models establishes the robustness of this finding. The decision tree model provides a potential framework for how oximetry (ODI3) and PTTrs could be used together to help diagnose OSA in children [Figure A2 (Appendix)].

Pitson et al. first demonstrated the value of PTTrs as a measure of respiratory effort in a study of eight patients with OSA treated with nasal CPAP, about 30 years ago (12). The patients aged 13–68 years were assessed with PTTrs, PTT-AI, oesophageal manometry, infra-red video, sound, electroencephalograms (EEG), oximetry, and pulse rate. The authors found an excellent correlation between the size of the swings in oesophageal pressure and the size of the PTT swings (mean *r* = 0.94). The authors concluded that PTTrs, a measure of respiratory oscillations in pulse transit time, is a potentially useful non-invasive measure of respiratory effort in patients with SDB.

Most subsequent studies investigating PTT in children with SDB have evaluated the PTT arousal index (4–8). Brietzke et al. studied 59 children with symptoms of SDB routinely scheduled for PSG, 11 of whom had previously had an adenotonsillectomy and 15 had a craniofacial syndrome (trisomy 21 and Apert syndrome) (6). A PTT-AI threshold of 7.4 events/h was found to identify OSA [defined as apnoea-hypopnoea index (AHI) > 3], with sensitivity 0.93 and specificity 0.91. A PTT-AI threshold of 5.4 events/h identified OSA (defined as AHI > 1), with sensitivity 0.81 and specificity 0.76.

Bradley et al. also assessed PTT as a surrogate measure of AHI in 51 children with suspected SDB undergoing PSG (7). The authors found a PTT-AI threshold of 11.36 events/h identified OSA (defined as AHI >3) with a sensitivity of 0.94 and specificity of 0.62. The same PTT-AI cut-off identified OSA (defined as AHI >1) with a sensitivity of 0.66 and a specificity of 0.67. Both studies suggest that PTT-AI may be a good screening test for moderate to severe OSA (AHI ≥ 3); however, the studies were not able to show that PTT-AI is reliable in differentiating between OSA and primary snoring.

Mehendale et al. used the Visilab sleep system, incorporating PTT inspiratory effort (PTTrs), in the pre- and postoperative evaluation of 44 children with velopharyngeal incompetence who underwent either a Sommerlad palate repair or Hynes pharyngoplasty (11). The authors found a significant increase in PTT swing (*p* = 0.04) in children who underwent Hynes pharyngoplasty, reflecting increased upper airway resistance and increased respiratory effort in those who had this procedure. They also found a significant increase in the OSA/hypopnea grading in children who had the Hynes pharyngoplasty. Their findings suggest that PTTrs detects increased respiratory effort associated with increased upper airway resistance, which the authors conclude makes it especially useful for detecting mild OSA.

We previously reported results of an observational study of 368 children with SDB, in which we found PTTrs of 17.92 ms identified children with OSA/UARS with sensitivity of 0.8 [Confidence intervals (CI): 0.62–0.90] and specificity 0.79 [0.49–0.87] using a limited multi-channel sleep study (oximetry, heart rate, movement, video, sound) as the comparator (9). A cut-off of 20.14 ms had a sensitivity of 0.61 [0.46–0.76] and specificity of 0.91 [0.81–0.97]. A PTT-AI of 16.06/h identified UARS/OSA with sensitivity 0.85 [0.67–0.92] and specificity 0.37 [0.17–0.48]. The findings demonstrate the limitations of PTT-AI in clinical practise due to its poor specificity. The PTTrs findings, however, suggest it may have utility in the diagnosis of OSA and may detect children with clinically significant obstruction with reasonable accuracy. These findings require validation in a study comparing PTTrs with PSG.

A systematic review of the diagnostic accuracy of oximetry in a mixed population of children (with and without comorbidities) with SDB shows that oximetry detects OSA with a sensitivity of 0.82 [0.76, 0.87] and a specificity of 0.75 [0.60, 0.85] (3). In children without comorbidities, oximetry detects OSA with a sensitivity of 0.77 [0.59, 0.90] and a specificity of 0.92 [0.36, 1.00]. In children with comorbidities (e.g., Trisomy 21, obesity, neuromuscular disorders) the sensitivity was 0.49 [0.31, 0.67] and specificity 0.87 [0.78, 0.93]. PSG definitions of OSA severity were as follows: AHI ≥ 1 < 5 (mild); AHI ≥ 5 < 10 (moderate); AHI ≥ 10 (severe).

It can therefore be anticipated that in a mixed population of 100 children with a PSG-confirmed diagnosis of OSA (defined as AHI ≥ 1), oximetry would not identify about 20 children with OSA. In this study, we identified 84 children with OSA using MCSS, based on accepted oximetry criteria. Therefore, we could reasonably assume that if we used PSG to evaluate our cohort, we could expect to identify an additional 25–30 children with OSA who were missed by oximetry. We note, however, that MCSS identifies an extra group of 132 children, whom we describe as having mild OSA/UARS, who have the same video evidence of an obstructed breathing pattern (an airway opening noise, and associated arousal) as the 84 children with oximetry-confirmed OSA. We surmise that some children with video evidence of an obstructed breathing pattern, but normal or inconclusive oximetry, would fulfil PSG criteria for mild OSA (defined as AHI ≥ 1 and < 5) because of the ability of PSG to detect hypopnoeas and cortical arousals with EEG, in children who have no oxygen desaturation. However, we think that many other children in the group of 132 will not meet the AASM-defined PSG criteria for OSA, but we presume that, based on the arousals witnessed on video, many of these children would meet criteria for UARS. We have therefore used a combined category of UARS/mild OSA in this study because of the limitations of MCSS in discriminating between the two categories.

Our use of MCSS instead of PSG is a significant limitation of this study. The lack of EEG recording reduces the ability of MCSS to accurately capture arousals; however, we have described how we use video to determine this instead, by analysing the heart rate, movement, and changes in the PTT traces to help identify sections of video that require careful review. Evidence to support the use of video in the diagnosis of SDB is provided in a study by Sivan et al., who investigated the diagnostic accuracy of a 30-min sleep video recording in 58 children with symptoms of SDB who also had PSG (19). The authors found that they could identify OSA with a sensitivity of 0.94 (95% CI: 0.81; 0.99) and specificity 0.68 [0.45; 0.86].

The only validation study of MCSS vs. PSG, to date, was conducted by Van Someren et al. The authors evaluated 10 children aged 2 months to 6 years and 4 months with both the Visilab system (oximetry, sound, video, and movement) and a conventional polysomnographic system (Oxcams), incorporating pulse oximetry, ECG, nasal airflow (thermistors), chest and abdominal movement (impedance), and video (20). There were just two discrepancies in the final diagnosis between the two systems. One child deemed to have a normal study with the Visilab system had a mild obstruction identified with PSG, whilst another deemed to have obstruction with the Visilab system was shown to have mixed apnoea with PSG. We are confident that children diagnosed with moderate or severe OSA using MCSS will correlate closely with PSG criteria, given the known specificity of oximetry for diagnosing OSA and the additional video confirmation of these findings (3). We acknowledge that the use of MCSS to diagnose UARS or mild OSA is based on limited evidence and requires further validation.

Allowing for the limitations of MCSS, we note that PTTrs appears able to identify children with clinically significant obstruction (mild OSA) who are detected with video but not with oximetry. It is this feature of PTTrs that we consider makes it a potentially valuable tool for detecting OSA/UARS in children. We acknowledge that further work is required to validate our assumptions about whether arousals identified on video correlate with those confirmed by PSG. We speculate that MCSS may underestimate the prevalence of true UARS, and furthermore, we suspect the specificity of mild OSA compared with PSG may be reduced due to our use of a combined category. We therefore anticipate that the specificity of PTTrs compared with PSG may be reduced, relative to these findings. However, we expect the sensitivity of detecting OSA with MCSS to be moderate or high. We presume that some children diagnosed with UARS using video criteria will benefit from intervention despite not meeting PSG criteria for OSA. Others will be safely managed with watchful waiting (16). We acknowledge the lack of consensus about which children with UARS require treatment, but note there is increased recognition of complications and the need to treat some children (21, 22).

A strength of MCSS is that it is likely to provide better accuracy than oximetry alone, which is currently the main modality available to clinicians in secondary care in the UK and many European countries. A significant advantage of PTT is that it can be easily obtained simultaneously with oximetry using the plethysmography probe and ECG leads, which are connected via a small box. This makes it potentially useful for home studies and increases its accessibility.

However, a significant limitation of PTT is its propensity to be affected by artefact, resulting in 31% of data being excluded from this study. Griffon et al. similarly found that a third of PTT data needed to be excluded in their study due to artefact (5). We acknowledge that our decision to accept a minimum of 3 h of artefact-free PTT data for analysis is based on limited data from our previous study, where we established this threshold for pragmatic purposes (9). Had we chosen to use a minimum of 4 h of artefact-free PTT data to align with the accepted minimum requirement for oximetry studies (3), we would have had to exclude a further 210 subjects from our dataset, which would have resulted in 50% of the total cohort being excluded due to PTT artefact. We have attempted to evaluate whether our decision to accept 3 h of artefact-free PTT data rather than 4 h had an impact on our findings by reanalysing the data, but only including children with a minimum of 4 h of artefact-free PTT data. Results of this re-analysis are shown in Table 10 and Figure 9. Table 10 shows that using a PTT duration of at least 4 h would result in the exclusion of 210 subjects uniformly across the diagnostic categories, with no group experiencing a relatively higher loss of data compared to another. Furthermore, logistic regression analysis using the two principal variables of ODI3 and PTTrs but only using data from children with a minimum duration of 4 h, results in no significant change in the diagnostic accuracy of the model (accuracy: 0.79; 95% CI: 0.75, 0.83) compared with our use of a PTT duration of 3 h or more (accuracy: 0.80, 95% CI: 0.77, 0.83). We conclude from this analysis that the use of 3 h of artefact-free PTT data is an acceptable minimum threshold for diagnostic purposes.

We acknowledge that a lack of inter-rater or intra-rater reliability data for the use of MCSS in determining the sleep study diagnosis is a limitation of our study and a potential source of bias. The authors were cognisant of the potential for reporting bias, and to minimise this, the reporting clinician adhered closely to our previously reported video and oximetry classification criteria and chose to remain blind to PTT data until after diagnostic categories had been determined (9). Having a physiologist supporting all sleep study reporting sessions provided additional oversight of the reporting clinician's adherence to agreed criteria. A future study needs to evaluate inter- and intra-rater consistency of MCSS reporting before our methods can be accepted into wider practise.

We did not use any validated questionnaires in our assessment of children in this study, but we do not consider that this has a material impact on the relevance of our findings. All children included in this study had been considered by an otolaryngologist, paediatrician, or general practitioner for concerns about sleep-disordered breathing and referred for assessment within a secondary care setting. The population being studied is therefore a subgroup of children who have a higher likelihood of obstructive sleep apnoea compared to the general population. A systematic review of the evidence evaluating the diagnostic accuracy of sleep questionnaires to identify children with obstructive sleep apnoea suggests they have moderate sensitivity and low specificity for diagnosing SDB in children (3). This contrasts with the diagnostic accuracy of oximetry, which we have discussed earlier. Given that our use of MCSS incorporates both oximetry and video, we surmise that MCSS has improved sensitivity and specificity compared to oximetry alone and offers much improved accuracy over the use of questionnaires. We therefore consider that not using a validated questionnaire in this study does not have a material impact on the findings.

We propose that a future study evaluates whether combining PTTrs and oximetry criteria can improve the detection of OSA. We believe that these two measurements can be easily obtained in children assessed in hospitals not equipped with state-of-the-art sleep diagnostic tools (i.e. PSG). Their use could enable clinicians to identify children with OSA more accurately without the need for PSG. PTTrs may be particularly useful in children with comorbidities in whom oximetry alone has low sensitivity for detecting OSA. We have previously demonstrated how the increased use of MCSS to obtain an accurate diagnosis of OSA facilitates conservative management and the potential for cost savings, whilst ensuring that children most in need of treatment receive it (16, 23).

## Conclusion

We have shown in a large sample of children with symptoms of SDB that those with mild OSA have a mean PTTrs of 20.7 ms. We also found a stepwise increase in PTTrs with increasing severity of sleep-disordered breathing. Machine learning analysis suggests that the oximetry index (ODI3) and PTTrs are the two most important predictors of OSA or UARS in our data. We conclude that using PTTrs alongside oximetry could improve the detection of OSA in children in centres with limited or no access to PSG. A validation study comparing PTTrs with PSG is needed to establish its usefulness.

## Summary

The effects of obstructive sleep apnoea (OSA) in children include impaired school performance, behaviour difficulties, and tiredness, amongst other things. Establishing a diagnosis requires a sleep study, but the best test, polysomnography (PSG), is expensive, complicated, and only available in a limited number of specialised centres. Oximetry is a less accurate but simpler test used in many centres and can be easily done at home. Oximetry is quite effective at identifying children with moderate or severe OSA, but cannot detect many children with mild OSA.

We have evaluated a test called pulse transit time (PTT) which is obtained using an oximetry probe on a finger or toe and ECG chest leads. We have studied a PTT measurement (PTTrs), about which there has been very little research to date, and found it much better at identifying children with OSA than the previously studied PTT arousal index.

We believe that PTTrs may detect children with mild OSA with reasonable accuracy; however, our findings require confirmation through a comparative study of PTTrs vs. PSG. We believe the best use of PTTrs will be in combination with oximetry and could help identify many more children with OSA without the need for PSG.

## Data Availability

The raw data supporting the conclusions of this article will be made available by the authors, without undue reservation.

## References

[1] Kaditis AG Alonso Alvarez ML Boudewyns A Alexopoulos EI Ersu R Joosten K. Obstructive sleep disordered breathing in 2-to-18-year old children: diagnosis and management. Eur Respir J. (2016) 47:69–94. 10.1183/13993003.00385-2015 26541535

[2] ENT UK. Safe Delivery of Paediatric ENT Surgery in the UK: A National Strategy. A Report of a Combined Working Party of the British Association for Paediatric Otolaryngology (BAPO), ENT UK, the Royal College of Anaesthetists (RCOA) and the Association of Paediatric Anaesthetists of Great Britain and Ireland (APAGBI) (2019). Available online at: https://www.entuk.org/_userfiles/pages/files/safe_delivery_paediatric_ent.pdf (Accessed September 3, 2025).

[3] Evans HJ Gibson NA Bennett J On behalf of the BTS Paediatric Sleep Disorders Guideline Development Group. British Thoracic society guideline for diagnosing and monitoring paediatric sleep-disordered breathing. Thorax. (2023) 78:s1–27. 10.1136/thorax-2022-218938 37295792

[4] Katz ES Lutz J Black C Marcus CL. Pulse transit time as a measure of arousal and respiratory effort in children with sleep-disordered breathing. Pediatr Res. (2003) 53:580–8. 10.1203/01.PDR.0000057206.14698.47 12612196

[5] Griffon L Amaddeo A Olmo Arroyo J Tenconi R Caggiano S Khirani S. et al. Pulse transit time as a tool to characterize obstructive and central apneas in children. Sleep Breath. (2018) 22:311–6. 10.1007/s11325-017-1488-3 28281031

[6] Brietzke SE Katz ES Roberson DW. Pulse transit time as a screening test for pediatric sleep-related breathing disorders. Arch Otolaryngol Head Neck Surg. (2007) 133: 980–984. 10.1001/archotol.133.10.980 17938320

[7] Bradley J Galland BC Bakker JP Tan E Gray A Taylor BJ. Pulse transit time and assessment of childhood sleep disordered breathing. Arch Otolaryngol Head Neck Surg. (2012) 138:398–403. 10.1001/archoto.2012.86 22508624

[8] Smith LA Dawes PJ Galland BC. The use of pulse transit time in pediatric sleep studies: a systematic review. Sleep Med Rev. (2018) 37:4–13. 10.1016/j.smrv.2016.11.006 28159487

[9] Yanney MP Prayle AP Rowbotham NJ Kurc M Tilbrook S Ali N. Observational study of pulse transit time in children with sleep disordered breathing. Front Neurol. (2020) 11:316. 10.3389/fneur.2020.00316 32457689 PMC7225317

[10] Smith S Yanney MP Prayle AP Jahnke N Rowbotham NR. Pulse Transit Time Respiratory Swing (PTTrs) as a Diagnostic Test for Obstructive Sleep Apnoea (OSA) in Children – A DIAGNOSTIC Test Accuracy Review. PROSPERO (2025). Available online at: https://www.crd.york.ac.uk/PROSPERO/view/CRD420251030385 (Accessed September 3, 2025).

[11] Mehendale FV Lane R Laverty A Dinwiddie R Sommerlad BC. Effect of palate re-repairs and hynes pharyngoplasties on pediatric airways: an analysis of preoperative and postoperative cardiorespiratory sleep studies. Cleft Palate Craniofac J. (2013) 50:257–67. 10.1597/11-198 22551554

[12] Pitson DJ Sandell A van den Hout R Stradling JR. Use of pulse transit time as a measure of inspiratory effort in patients with obstructive sleep apnoea. Eur Respir J. (1995) 8:1669–74. 10.1183/09031936.95.08101669 8586120

[13] Tasker C Crosby JH Stradling JR. Evidence for persistence of upper airway narrowing during sleep, 12 years after adenotonsillectomy. Arch Dis Child. (2002) 86:34–7. 10.1136/adc.86.1.34 11806880 PMC1719063

[14] Sidey-Gibbons J Sidey-Gibbons C. Machine learning in medicine: a practical introduction. BMC Med Res Methodol. (2019) 19:64. 10.1186/s12874-019-0681-4 30890124 PMC6425557

[15] Caffo B Diener-West M Punjabi NM Samet J. A novel approach to prediction of mild obstructive sleep disordered breathing in a population-based sample: the Sleep Heart Health Study. Sleep. (2010) 33:1641–8. 10.1093/sleep/33.12.1641 21120126 PMC2982734

[16] Yanney MP Rowbotham NJ Ng C Zulkifli M Shehata A Chidambaram A. Retrospective review of treatment outcomes and costs in children with sleep disordered breathing assessed with multi-channel studies. Sleep Med. (2024) 7:100115. 10.1016/j.sleepx.2024.100115 39022329 PMC11252078

[17] Horwood L Brouillette RT McGregor CD Manoukian JJ Constantin E. Testing for pediatric obstructive sleep apnea when health care resources are rationed. JAMA Otolaryngol Head Neck Surg. (2014) 140:616–23. 10.1001/jamaoto.2014.778 24851855

[18] Department of Health. Governance Arrangements for Research Ethics Committees: 2020 Edition (2023). Available online at: https://www.hra.nhs.uk/planning-and-improving-research/policies-standards-legislation/governance-arrangement-research-ethics-committees/ (Accessed September 3, 2025).

[19] Sivan Y Kornecki A Schonfeld T. Screening obstructive sleep apnoea syndrome by home videotape recording in children. Eur Respir J. (1996) 9:2127–31. 10.1183/09031936.96.09102127 8902478

[20] van Someren V Burmester M Alusi G Lane R. Are sleep studies worth doing? Arch Dis Child. (2000) 83:76–81. 10.1136/adc.83.1.76 10869008 PMC1718385

[21] Guilleminault C Khramtsov A. Upper airway resistance syndrome in children: a clinical review. Semin Pediatr Neurol. (2001) 8:207–15. 10.1053/spen.2001.29045 11768783

[22] Guilleminault C Los Reyes VD. Upper-airway resistance syndrome. Handb Clin Neurol. (2011) 98:401–9. 10.1016/B978-0-444-52006-7.00026-5 21056201

[23] Yanney MP Rowbotham NJ Ng C Zulkifli M Shehata A Chidambaram A. Prospective evaluation of the impact of multi-channel studies on treatment outcomes in children with sleep disordered breathing. Sleep Med. (2024) 7:100111 10.1016/j.sleepx.2024.100111 38800098 PMC11127274

