# Supplementary material for: Pulse transit time respiratory swing as a diagnostic test for obstructive sleep apnoea in children—An observational study
Source: Front Neurol. 2025 Sep 22;16:1632919. doi: 10.3389/fneur.2025.1632919 (PMC12497600; doi:10.3389/fneur.2025.1632919)
Supplement: Supplementary file 1 [file Supplementary_file_1.docx]

# APPENDIX

**Multinomial regression**

- Diagnostics and model fit: Separate logit models are run to detect outliers or influential data points and use the diagnostic tools on each model.
- Sample size: Multinomial regression uses a maximum likelihood estimation method; it requires a large sample size. It also uses multiple equations. This implies that it requires an even larger sample size than ordinal or binary logistic regression.
- Complete or quasi-complete separation: Complete separation means that the outcome variable separates a predictor variable completely, leading to perfect prediction by the predictor variable.
- Perfect prediction means that only one value of a predictor variable is associated with only one value of the response variable. It is usually obvious from the output of the regression coefficients that something is wrong. A two-way tabulation of the outcome variable with the problematic variable can then be done to confirm this and then rerun the model without the problematic variable.
- Empty or small cells: Empty or small cells need to be checked by cross-tabulating categorical predictors and the outcome variable. If a cell has very few cases (a small cell), the model may become unstable, or it might not even run at all.

**Figure A: Example of a full study PTT montage with artefact highlighted for exclusion from analysis**


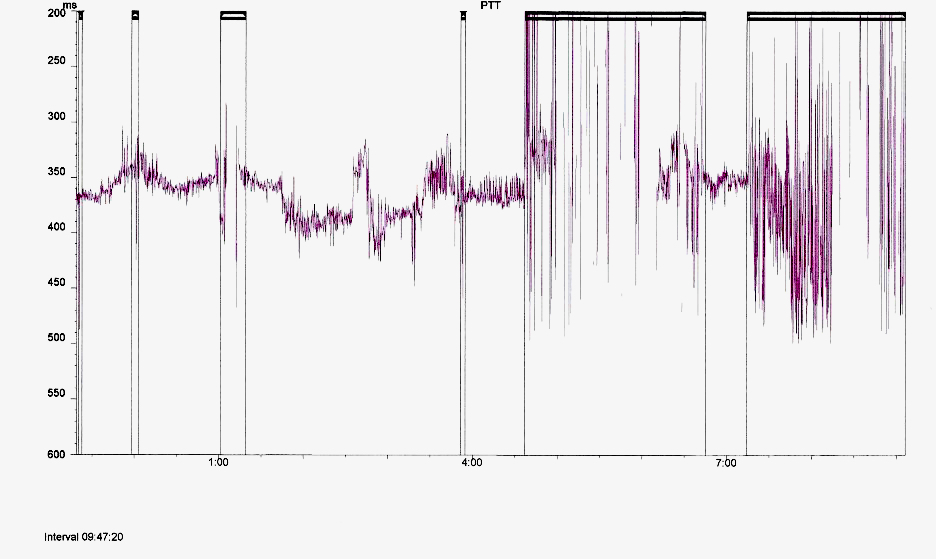


**Table A1: Results of Logistic Regression Model for Iteration 1**

**Table A2: Statistics showing the diagnostic performance of Logistic Regression**

**and a Random Forest plot obtained using a model with 6 variables**

| **=== Logistic Regression ===**  Confusion Matrix and Statistics  Reference  Prediction 0 1  0 114 22  1 8 42  AUC: 0.9081711  Accuracy: 0.8387  95% CI: (0.7778, 0.8885)  No Information Rate: 0.6559  P-Value [Acc > NIR]: 2.029e-08  Kappa: 0.6231  Mcnemar's Test P-Value: 0.01762  Sensitivity: 0.9344  Specificity: 0.6562  Pos Pred Value: 0.8382  Neg Pred Value: 0.8400  Prevalence: 0.6559  Detection Rate: 0.6129  Detection Prevalence: 0.7312  Balanced Accuracy: 0.7953  'Positive' Class: 0 |  | **=== Random Forest ===**  Confusion Matrix and Statistics  Reference  Prediction 0 1  0 117 24  1 5 40  AUC: 0.8860784  Accuracy: 0.8441  95% CI: (0.7838, 0.893)  No Information Rate: 0.6559  P-Value [Acc > NIR]: 7.235e-09  Kappa: 0.6284  Mcnemar's Test P-Value: 0.0008302  Sensitivity: 0.9590  Specificity: 0.6250  Pos Pred Value: 0.8298  Neg Pred Value: 0.8889  Prevalence: 0.6559  Detection Rate: 0.6290  Detection Prevalence: 0.7581  Balanced Accuracy: 0.7920  'Positive' Class: 0 |
| --- | --- | --- |

**Figure B: Comparison Receiver Operator Characteristic curves obtained using Logistic Regression and a Random Forest plot showing diagnostic performance of the model with 6 variables**


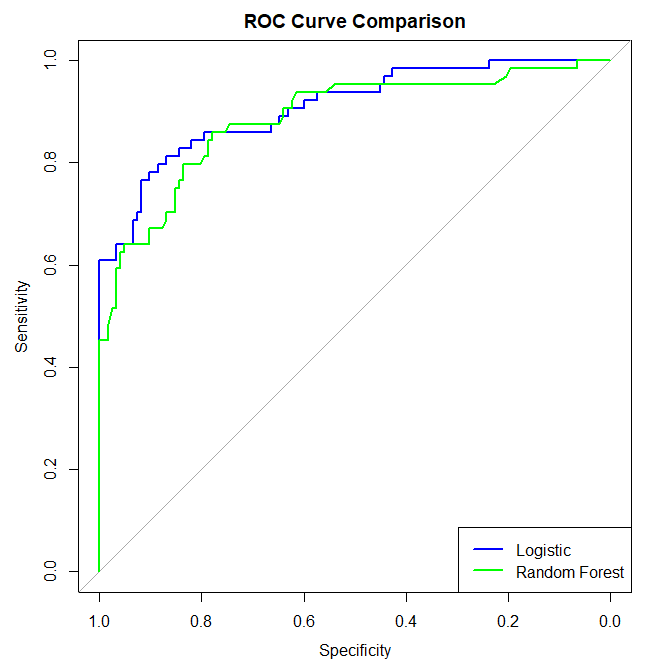


*Data was split into training and test sets and results are presented from the test set*

**Figure C: Decision tree-based machine learning model using ODI3 and PTTrs**


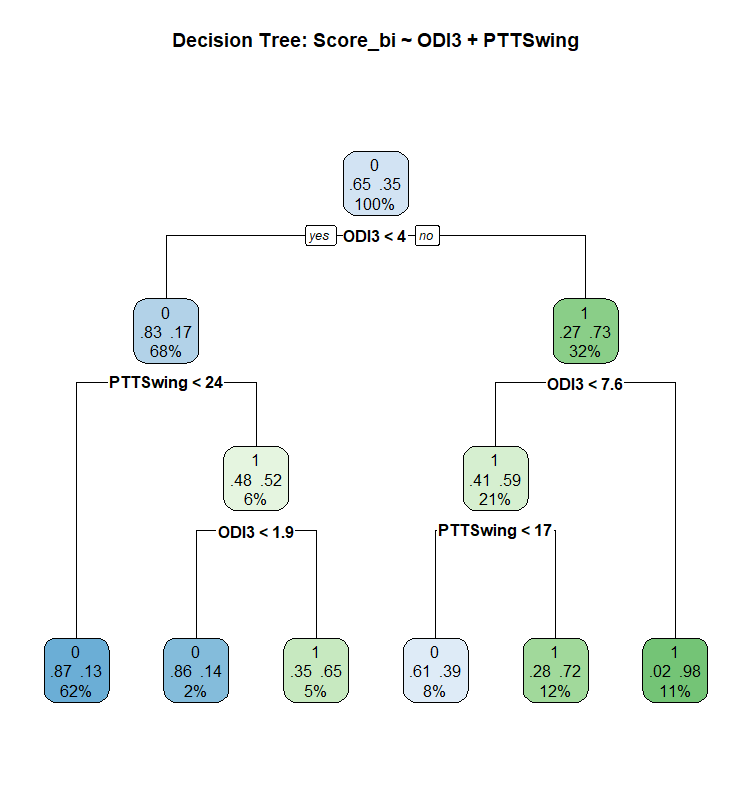


*Data was split into training and test sets and results are presented from the test set*

**Summary of interpretation of decision tree**

1. Start by looking at the ODI3 values: if it is less than 4, the patient has a 68% chance of NOT having OSA. If ODI3 value is greater than 4, the patient has a 32% chance of having OSA.
2. In the second step, if the patient’s ODI3 was greater than 4, you would then ask yourself if the ODI3 value is less than 7.6. If ODI3 is less than 7.6, then there is a 21% probability of having OSA. You would explore this further by looking at the PTTrs value. If PTTrs is less than 17, then the patient has an 8% probability of not having OSA. If PTTrs is greater than 17, they have a 12% probability of having OSA.

**Confusion Matrix and Statistics**

Reference

Prediction 0 1

0 111 25

1 11 39

Accuracy: 0.8065

95% CI: (0.7423, 0.8606)

No Information Rate: 0.6559

P-Value [Acc > NIR]: 4.553e-06

Kappa: 0.5477

Mcnemar's Test P-Value: 0.03026

Sensitivity: 0.9098

Specificity: 0.6094

Pos Pred Value: 0.8162

Neg Pred Value: 0.7800 Prevalence: 0.6559

Detection Rate: 0.5968

Detection Prevalence: 0.7312

Balanced Accuracy: 0.7596

'Positive' Class: 0
